# Risk factors for Bell’s palsy based on the Korean National Health Insurance Service National Sample Cohort data

**DOI:** 10.1038/s41598-021-02816-9

**Published:** 2021-12-03

**Authors:** Junhui Jeong, So Ra Yoon, Hyunsun Lim, Jangwon Oh, Hyun Seung Choi

**Affiliations:** 1 Department of Otorhinolaryngology, National Health Insurance Service Ilsan Hospital, 100 Ilsan-ro, Ilsandong-gu, Goyang, 10444 Korea; 2 Research and Analysis Team, National Health Insurance Service Ilsan Hospital, Goyang, Korea

**Keywords:** Diseases, Medical research, Risk factors

## Abstract

The associations between hypertension, diabetes, and dyslipidemia with Bell’s palsy have been controversial and only a few studies have assessed risk factors for Bell’s palsy based on population-based data. The aim of the present study was to evaluate whether sociodemographic factors such as sex, age, residence, household income, and metabolic diseases such as hypertension, diabetes, and dyslipidemia were risk factors for Bell’s palsy using the National Health Insurance Service National Sample Cohort data of Korea. Patients who visited an outpatient clinic twice or more or had one or more admission and received steroid medication under the International Classification of Diseases diagnostic codes for Bell’s palsy from 2006 to 2015 were defined as patients with Bell’s palsy in this study. The associations between sociodemographic factors and metabolic diseases to Bell’s palsy were analyzed with univariate and multivariate Cox proportional hazard regression models. There were 2708 patients with Bell’s palsy recorded from 2006 to 2015. Male sex, advanced age, residence in a location other than the capital and metropolitan cities, hypertension, and diabetes were significant risk factors for Bell’s palsy. This study is significant for patients and providers because we analyzed the relationships using a population-based database over a long-term follow-up period.

## Introduction

Bell’s palsy is an acute idiopathic paresis or paralysis of the peripheral facial nerve that contracts muscles of the face^1–8^. It is the most common cause of peripheral facial palsy^4,9^. The annual incidence of Bell’s palsy has been reported to range from 11.5 to 53.3 per 100,000^3,10,11^. The etiology of Bell’s palsy remains unclear^2,5,7,12–14^; however, it has been attributed to the reactivation of latent herpes simplex virus type 1 (HSV-1) infection in the geniculate ganglion^4,7,12,14^. Ischemia and autoimmune conditions have also been proposed as causes^2,10,13,14^.

Several factors associated with Bell’s palsy have been reported, including hypertension, diabetes, and dyslipidemia, which are associated with microangiopathy and can be risk and prognostic factors for Bell’s palsy^13^. These are characteristic of metabolic syndrome, which is a chronic metabolic disorder^10,15^. Metabolic syndrome has been reported to be associated with a worse recovery rate in Bell’s palsy^10^. Although several reports have indicated that there is an association between hypertension, diabetes, and dyslipidemia and Bell’s palsy, to date the reports remain controversial^1,8,9,13,16^.

A few studies have assessed risk factors for Bell’s palsy based on population-based data. Considering the controversial and uncertain relationship, we used population-based national sample cohort data from Korea to evaluate whether sociodemographic factors such as sex, age, residence, household income, and metabolic diseases such as hypertension, diabetes, and dyslipidemia were risk factors for Bell’s palsy.

## Methods

### Subjects

This retrospective study included public data from the National Health Insurance Service National Sample Cohort data of Korea, which is a 2% representative subsample of the entire National Health Insurance Service data. It was established with systematic stratified random sampling with proportional allocation within each stratum, resulting in the representativeness of the whole data. All Korean citizens are required to join the National Health Insurance Service; thus, the results of this study are based on the whole population in Korea.

Patients who visited the outpatient clinic twice or more or had one or more admission from 2006 to 2015 and received steroid medication under the diagnostic codes for Bell’s palsy in the International Classification of Diseases (ICD) (G51.0) were defined as patients with Bell’s palsy. Thus, steroid treatment was performed for all members of the patient group. The control group was established using the rest of the patients from the database. Considering the easy accessibility to medical services due to the mandatory National Health Insurance Service in Korea, as well as the large size of the control group, patients with Bell’s palsy who had not visited the hospital in the control group were thought to be negligible. The patients with Bell’s palsy and control groups were divided according to sociodemographic factors such as sex, age, residence, and household income, and metabolic diseases such as hypertension, diabetes, and dyslipidemia, that were diagnosed during 2003 to 2015, prior to the onset of Bell’s palsy. Patients with hypertension, diabetes, and dyslipidemia were defined using diagnostic codes in the ICD (I10-15 for hypertension; E10-14 for diabetes; E78 for dyslipidemia) and relevant medication claim codes including beta blockers, alpha blockers, calcium channel blockers, angiotensin converting enzyme inhibitors, angiotensin II receptor blockers, diuretics for hypertension, insulin, biguanide, alpha-glucosidase inhibitors, sodium glucose co-transporter-2 inhibitors, sulfonylurea, meglitinide, thiazolidinedione, dipeptidyl peptidase-4 inhibitor for diabetes, statin, ezetimibe, niacin, fibrate, cholestyramine, and omega-3 fatty acid for dyslipidemia.

### Data analysis

First, the incidence and sociodemographic distribution of patients with Bell’s palsy were investigated because they were fundamental to the analysis of risk factors. Associations between sociodemographic factors such as sex, age, residence, household income, and metabolic diseases such as hypertension, diabetes, and dyslipidemia with Bell’s palsy were analyzed. Data from 2006 to 2015 were included in the univariate and multivariate Cox proportional hazard regression models. Statistical analyses were performed using SAS 9.4 (SAS Institute, Cary, NC, USA). In all analyses, *p* < 0.05 was considered statistically significant.

### Ethical approval

This study was in accordance with the 1964 Helsinki declaration and its later amendments or comparable ethical standards. The Institutional Review Board of the National Health Insurance Service Ilsan Hospital approved this study (NHIMC 2020-10-009). All methods were performed in accordance with the relevant guidelines and regulations. Written informed consent was waived by the Institutional Review Board of the National Health Insurance Service Ilsan Hospital because of the retrospective nature of the study.

## Results

There were 2,708 patients with Bell’s palsy from 2006 to 2015. The average annual incidence of Bell’s palsy was 25.9 per 100,000 for 10 years (Table 1). The sociodemographic distributions of patients with Bell’s palsy from 2006 to 2015 are presented in Table 2.

**Table 1:** The number of patients and incidence of Bell’s palsy from 2006 to 2015 based on the National Sample Cohort data of Korea.

Year	Number of patients with Bell's palsy	Total number of people in the National Sample Cohort	Incidence (per 100,000)
2006	215	1,021,208	21.1
2007	213	1,031,653	20.6
2008	257	1,035,089	24.8
2009	263	1,038,462	25.3
2010	260	1,042,706	24.9
2011	279	1,046,465	26.7
2012	332	1,050,743	31.6
2013	276	1,053,952	26.2
2014	301	1,057,454	28.5
2015	312	1,061,141	29.4
Total	2708	10,438,873	25.9

**Table 2:** Sociodemographic distribution of patients with Bell’s palsy. ^†^The sum of patients in household income is less than the total due to missing values.

	Bell’s palsy (n = 2708)	
	n	%
**Sex**		
Male	1422	52.5
Female	1286	47.5
**Age (years)**		
≤ 29	617	22.8
30–49	1035	38.2
50–69	840	31.0
≥ 70	216	8.0
**Residence**		
Seoul (capital)	508	18.8
Metropolitan cities	674	24.9
Other	1526	56.4
**Household income**^**†**^		
1st quintile (lowest)	380	14.7
2nd quintile	426	16.5
3rd quintile	472	18.3
4th quintile	604	23.4
5th quintile (highest)	697	27.0

Univariate Cox proportional hazard regression analysis indicated that significant risk factors for Bell’s palsy were male sex (hazard ratio [HR] = 1.105, 95% confidence interval [CI] = 1.025– 1.191), advanced age (HR = 1.759, 95% CI = 1.560–1.984 [30–39 years old]; HR = 2.231, 95% CI = 1.991–2.500 [40–49 years old]; HR = 2.853, 95% CI = 2.531–3.217 [50–59 years old]; HR = 3.447, 95% CI = 3.029–3.922 [60–69 years old]; HR = 3.33, 95% CI = 2.818–3.935 [70–79 years old]; HR = 2.886, 95% CI = 2.079–4.006 [80 years old or older]), residence in regions other than the capital and metropolitan cities (HR = 1.181, 95% CI = 1.068–1.306), hypertension (HR = 2.286, 95% CI = 2.075–2.519), diabetes (HR = 2.743, 95% CI = 2.378–3.164), and dyslipidemia (HR = 1.875, 95% CI = 1.570–2.239).

Multivariate Cox proportional hazard regression analysis indicated that significant risk factors for Bell’s palsy were male sex (HR = 1.169, 95% CI = 1.081–1.263), advanced age (HR = 1.730, 95% CI = 1.530–1.955 [30–39 years old]; HR = 2.153, 95% CI = 1.915–2.420 [40–49 years old]; HR = 2.554, 95% CI = 2.248–2.901 [50–59 years old]; HR = 2.801, 95% CI = 2.422–3.238 [60–69 years old]; HR = 2.663, 95% CI = 2.204–3.217 [70–79 years old]; HR = 2.278, 95% CI = 1.576–3.293 [80 years old or older]), residence in regions other than the capital and metropolitan cities (HR = 1.182, 95% CI = 1.067–1.309), hypertension (HR = 1.362, 95% CI = 1.208–1.535), and diabetes (HR = 1.579, 95% CI = 1.347–1.851) (Table 3).

**Table 3:** Risk factors for Bell’s palsy based on univariate and multivariate Cox proportional hazard regression analyses.

					Univariate analysis				Multivariate analysis^†^			
	Bell's palsy (n = 2,708)	%	Control (n = 1,028,953)	%	HR	95% CI		*p*-value	HR	95% CI		*p*-value
**Sex**												
Male	1422	52.5	515,709	50.1	1.105	1.025	1.191	0.0096*	1.169	1.081	1.263	< 0.0001*
Female	1286	47.5	513,244	49.9	1				1			
**Age (years)**												
≤ 29	617	22.8	421,388	41	1				1			
30–39	467	17.3	181,785	17.7	1.759	1.560	1.984	< 0.0001*	1.730	1.530	1.955	< 0.0001*
40–49	568	21	175,350	17	2.231	1.991	2.500	< 0.0001*	2.153	1.915	2.420	< 0.0001*
50–59	472	17.4	115,309	11.2	2.853	2.531	3.217	< 0.0001*	2.554	2.248	2.901	< 0.0001*
60–69	368	13.6	77,278	7.5	3.447	3.029	3.922	< 0.0001*	2.801	2.422	3.238	< 0.0001*
70–79	178	6.6	43,229	4.2	3.33	2.818	3.935	< 0.0001*	2.663	2.204	3.217	< 0.0001*
≥ 80	38	1.4	14,614	1.4	2.886	2.079	4.006	< 0.0001*	2.278	1.576	3.293	< 0.0001*
**Residence**												
Seoul (capital)	508	18.8	213,921	20.8	1				1			
Metropolitan cities	674	24.9	267,152	26	1.064	0.849	1.194	0.2884	1.068	0.948	1.201	0.2733
Others	1526	56.4	547,880	53.3	1.181	1.068	1.306	0.0011*	1.182	1.067	1.309	0.0014*
**Household income**^**‡**^												
1st quintile (lowest)	380	14.7	144,996	14.8	1				1			
2nd quintile	426	16.5	160,304	16.3	1.009	0.879	1.158	0.9009	1.061	0.924	1.219	0.4021
3rd quintile	472	18.3	178,183	18.2	1.004	0.877	1.149	0.9588	1.056	0.922	1.209	0.4286
4th quintile	604	23.4	239,254	24.3	0.958	0.843	1.089	0.5137	0.990	0.871	1.126	0.8833
5th quintile highest)	697	27	258,850	26.4	1.026	0.905	1.162	0.6891	1.009	0.890	1.144	0.8919
**Hypertension**												
No	2205	81.4	928,701	90.3	1				1			
Yes	503	18.6	100,252	9.7	2.286	2.075	2.519	< 0.0001*	1.362	1.208	1.535	< 0.0001*
**Diabetes**												
No	2504	92.5	996,728	96.9	1				1			
Yes	204	7.5	32,225	3.1	2.743	2.378	3.164	< 0.0001*	1.579	1.347	1.851	< 0.0001*
**Dyslipidemia**												
No	2502	92.4	981,281	95.4	1				1			
Yes	206	7.6	47,672	4.6	1.875	1.570	2.239	< 0.0001*	1.017	0.842	1.229	0.8614

*HR* hazard ratio, *CI* confidence interval.

*p value < 0.05.

^†^All factors were adjusted in the multivariate Cox proportional hazard regression analysis. ^‡^The sum of patients in household income is less than the total due to missing values.

## Discussion

Several risk factors associated with Bell’s palsy have been reported to date. The American Academy of Otolaryngology-Head and Neck Surgery clinical practice guideline for Bell’s palsy indicates that patients that are pregnant, have severe preeclampsia, obesity, hypertension and chronic hypertension, diabetes, and upper respiratory ailments are at a higher risk for Bell’s palsy^3^.

Several reports have indicated that the distribution of Bell’s palsy is equal among men and women^2,7,12^. A Taiwanese population-based study reported that there were more female patients with Bell’s palsy (52.4%) than males (47.6%)^5^. However, there were more male patients with Bell’s palsy (52.5%) than females (47.5%) in our population-based study, and male sex was a significant risk factor for Bell’s palsy. Recently, Lee and Kim also reported that there were more male patients with Bell’s palsy than females and that male sex was also a risk factor for Bell’s palsy based on population-based data in Korea^11^.

Bell’s palsy has demonstrated a peak incidence in patients between 15 and 45 years of age^1,3,12^. However, Lee and Kim’s study, which was based on National Health Insurance Service data in Korea, showed a peak incidence of Bell’s palsy between 60 and 69 years of age^11^. In the study based on the Korean National Health and Nutrition Examination Survey, another population-based data set in Korea, advanced age was significantly associated with facial palsy, including Bell’s palsy^17^. Another epidemiologic study in Italy showed that advanced age was a significant risk factor for Bell’s palsy, and correlated with a linear trend when age increased^12^. In our study, the risk for Bell’s palsy increased when age increased and peaked at 60–69 years of age. The age of the general population is increasing, which can impact reactivation of latent herpes simplex virus infection in the geniculate ganglion and also the incidence of ischemia, which are more likely with old age, thus resulting in a high risk for Bell’s palsy for older patients compared with a peak incidence in a previous report.

To date, no studies have been conducted to verify the relationship between socioeconomic factors such as household income and residence with Bell’s palsy. In the present study, residence in a location other than the capital and metropolitan cities, which indicates small-sized cities or rural areas, was associated with significant risk factors for Bell’s palsy. It is hypothesized that the risk was higher due to the aging of the population in rural areas.

The association between hypertension and Bell’s palsy has been described in many studies. Savadi-Oskouei et al. reported that hypertension may increase the risk of Bell’s palsy in patients over 40 years of age^2^. In their systematic review, Jörg et al. reported that there might be an association between uncontrolled severe hypertension and peripheral facial palsy in children^18^. Hung et al. reported that hypertension was significantly higher in the Bell’s palsy group than in the control group in a population-based study in Taiwan^5^.

In contrast, several studies reported that there were no associations between hypertension and Bell’s palsy. Peitersen reported that Bell’s palsy is not more frequent in patients with hypertension than in those without hypertension^1^. Monini et al. reported that hypertension was not a significant risk factor for Bell’s palsy^12^. Riga et al. reported that hypertension was not associated with the severity of Bell’s palsy at initial diagnosis, nor was it associated with the recovery from Bell’s palsy after six months^13^. Additionally, Chang et al. reported that hypertension was not associated with facial palsy, including Bell’s palsy with House-Brackmann grade 3–6, whereas cardiovascular disease was associated with those clinical indicators^17^.

In our study, hypertension was confirmed to be a risk factor for Bell’s palsy; Bell’s palsy and hypertension was defined by the diagnostic and medication claim codes. Furthermore, our study analyzed population-based data over a long-term period from 2006 to 2015. Thus, based on the long-term data, hypertension has been confirmed as a risk factor for Bell’s palsy. However, the etiology is unclear, as small hemorrhages in the facial canal, vascular lesions, and partial necrosis in the nerve may cause facial palsy^2,7,16^. Hematoma or thrombus caused by hypertension in the facial canal could compress the facial nerve. Thickened vessel and perineural edema due to hypertension could also compress the facial nerve^10^. Of note, uncontrolled hypertension is particularly associated with Bell’s palsy^7^. Patients with uncontrolled hypertension have a higher possibility of hemorrhage into the facial canal and necrosis in the facial nerve than those with controlled hypertension, which likely contributes to these findings.

Many studies have reported the association between diabetes and Bell’s palsy. Savadi-Oskouei et al. reported that diabetes was a predictor of Bell’s palsy^2^. Riga et al. reported that diabetes with abnormal glycosylated hemoglobin (Hb1A1c) was associated with the severity of Bell’s palsy at initial diagnosis^13^. Hung et al. reported that diabetes was significantly higher in the Bell’s palsy group than in the control group in a population-based study in Taiwan^5^.

In contrast, Monini et al. reported that diabetes was not a significant risk factor for Bell’s palsy^12^. Sevik Elicora and Erdem reported that diabetes was not associated with the severity and recovery rate in Bell’s palsy^9^. Chang et al. reported that diabetes was not associated with facial palsy, including Bell’s palsy with House-Brackmann grade 3–6^17^. However, our study revealed that diabetes was an associated risk factor for Bell’s palsy.

Microangiopathy such as vascular insufficiency and diabetic polyneuropathy were associated with a poor prognosis^1,7,8,14^. A vascular rather than metabolic mechanism has been suggested for facial palsy in diabetes^2^. Chronic nerve ischemia can occur due to the reduction in endoneurial oxygen, reduced blood flow in the nerve, and epineurial arteriovenous shunt^13^. Consequently, microangiopathy may be a compensatory response to endoneurial ischemia or hypoxia. In addition, hyperglycemia induces direct nerve injury due to increased oxidative stress, accumulation of advanced glycation end products, impaired axonal transport, and impaired flow through the polyol pathway^10,13^.

Several studies have described an association between dyslipidemia and Bell’s palsy. Hung et al. reported that hyperlipidemia was significantly higher in the Bell’s palsy group than in the control group in their population-based study in Taiwan. Regular statin (3-hydroxy-3-methylglutaryl coenzyme A reductase inhibitor) use (≥ 60 days within 6 months) was significantly associated with Bell’s palsy^5^.

In contrast, Riga et al. reported that hypercholesterolemia was not associated with the severity of Bell’s palsy at initial diagnosis nor was it associated with the recovery from Bell’s palsy after six months^13^. Chang et al. found that hyperlipidemia was not associated with facial palsy, including Bell’s palsy with House-Brackmann grade 3–6^17^.

Kim et al. reported no relationship between previous statin use over the course of 2 years and the incidence of Bell’s palsy in patients 40 years of age or older, based on population-based data in Korea, although there were significantly more patients with dyslipidemia in the Bell’s palsy group than in control group^6^. Because we defined patients with dyslipidemia using diagnostic codes and relevant medication claim codes such as statins, our results indicated that dyslipidemia with statin use may not be a risk factor for Bell’s palsy, which is in agreement with the report by Kim et al.

Triglyceride and high-density lipoprotein-C are associated with atherosclerosis. Microcirculatory disorders caused by atherosclerosis increase the accumulation of lipids and cholesterol in the vessel, which induces endothelial dysfunction, plaque formation, vascular inflammation and obstruction, microvascular ischemia, and neuropathy^10^.

There are several possible etiologies for the association between statin use and Bell’s palsy. The potential risk of peripheral neuropathy due to statin use has previously been reported^5^. Statins can induce a disturbance in neurite outgrowth and maintenance and the neurotoxic effects of statins could increase the risk of Bell’s palsy^6^. Statins inhibit the synthesis of ubiquinone, which is essential for the mitochondrial respiratory chain, resulting in disruption to the energy supply to neurons^5,6^. Changes in neuronal cell membrane composition could influence protein interactions and neuronal functions. Axonal degeneration and segmental demyelination could be associated with neuropathy in statin use^6^. In addition, statins could stimulate pro-inflammatory responses and induce autoimmunity^5^.

In contrast, there is also a neuroprotective effect with statin use^5,6^. Anti-inflammatory and anti-oxidative effects of statins have also been reported. These neuroprotective, anti-inflammatory, and anti-oxidative effects could reduce the risk of Bell’s palsy^6^. However, the neuroprotective effect of statins may be restricted to neuron bodies, not to nerve sheaths, and statins might negatively impact oligodendrocytes and myelin formation^5^. In this respect, Bell’s palsy, which is a peripheral neuropathy, could increase due to statin use. However, considering previous studies that reported an association between peripheral neuropathy and statin use, these adverse effects of statins might be minimal^6^.

In the present study, metabolic diseases were defined using both diagnostic and medication claim codes, which was more accurate than a definition using only diagnostic codes. Based on the results of the present study, hypertension and diabetes are associated with Bell’s palsy, whereas dyslipidemia is still controversial regarding its association with Bell’s palsy when considering the administration of statin.

There were several limitations to our study. First, the risk factors for Bell’s palsy were analyzed based on the diagnostic codes and medication claim codes, which do not reflect the actual incidence of the disease. Second, only the predicting factors for Bell’s palsy were analyzed and the prognostic factors were not evaluated because the degree and prognosis of Bell’s palsy could not be verified in the population-based database that was based on diagnostic and medication claim codes. Nonetheless, this study analyzed the relationship between sociodemographic factors and major chronic metabolic diseases with Bell’s palsy comprehensively using population-based database over a long-term period, which contributes to clinical and socio-economic understanding of this condition. Although novel risk factors were not revealed in the present study, this study was performed using population-based data and suggests that patients with risk factors for Bell’s palsy should be carefully evaluated for prevention and clinical practice of Bell’s palsy.

## Conclusion

In summary, male sex, advanced age, residence in a location other than the capital and metropolitan cities, hypertension, and diabetes were significant risk factors for Bell’s palsy based on population-based data in Korea.

## Data Availability

All the data used in this study are available from the Korea National Health Insurance Service National Sample Cohort database.
